# Trajectory-Based Modeling Improves Long-Term Outcome Prediction Beyond 90-Day Assessment in ICU-Treated Intracerebral Hemorrhage

**DOI:** 10.31083/RN52441

**Published:** 2026-08-25

**Authors:** Qing Su, Xini Zhang, Baogui Wang, Liyun Zhu, Yufeng Li

**Affiliations:** ^1^Emergency Medicine Department, The First Affiliated Hospital of Jinan University, 510632 Guangzhou, Guangdong, China

**Keywords:** intracerebral hemorrhage, intensive care unit, recovery trajectories, prognostication, modified Rankin Scale, quality of life

## Abstract

**Objective:**

To identify distinct long-term functional recovery trajectories among intensive care unit (ICU)–treated patients with spontaneous intracerebral hemorrhage (ICH) and to evaluate whether trajectory-based classification improves prediction of long-term quality of life (QoL) compared with conventional single time-point functional assessment.

**Methods:**

We conducted a prospective longitudinal cohort study of consecutive adult patients with spontaneous ICH admitted to a tertiary ICU between January 2024 and January 2025. Survivors to hospital discharge were followed for 12 months. Functional status was assessed using the modified Rankin Scale (mRS) at hospital discharge, 90 days, 6 months, and 12 months. Latent class mixed modeling (LCMM) was used to identify distinct recovery trajectories. Health-related QoL at 12 months was measured using the EuroQol 5-Dimension 5-Level questionnaire (EQ-5D-5L) instrument. Multinomial logistic regression was performed to evaluate associations between early clinical characteristics and trajectory membership. Receiver operating characteristic curve analysis was conducted to compare the predictive performance of trajectory membership and 90-day mRS for 12-month QoL.

**Results:**

Among 196 patients (mean age 61.7 ± 12.4 years; 60.2% male), three distinct functional recovery trajectories were identified: good, moderate, and poor recovery. Twelve-month QoL demonstrated a graded decline across trajectory groups. Older age, lower admission Glasgow Coma Scale scores, and larger hematoma volume were independently associated with both moderate and poor recovery trajectories, while intraventricular hemorrhage, mechanical ventilation, and prolonged sedation (>48 hours) were specifically associated with the poor recovery trajectory. Trajectory-based classification demonstrated superior predictive performance for 12-month QoL compared with 90-day mRS (area under the curve [AUC], 0.81 vs 0.72; *p* = 0.003), with significant improvement in risk discrimination. Trajectory-based classification also improved risk reclassification (net reclassification improvement (NRI) = 0.27; integrated discrimination improvement (IDI) = 0.09).

**Conclusion:**

ICU-treated patients with intracerebral hemorrhage exhibit distinct long-term recovery trajectories that are strongly associated with quality of life. Importantly, trajectory-based classification provides incremental prognostic value beyond conventional single time-point functional assessment, supporting a shift toward dynamic outcome modeling for long-term prognostication in ICH.

## 1. Introduction

Intracerebral hemorrhage (ICH) is one of the most severe forms of stroke and remains a leading cause of death and long-term disability worldwide [1,2]. Although advances in acute neurocritical care have improved early survival, many patients who survive the initial hospitalization experience persistent neurological deficits, impaired functional independence, and reduced quality of life (QoL) [3,4]. As a result, accurate long-term prognostication has become a central challenge in contemporary ICH research.

Recovery after ICH is highly heterogeneous, and patients with similar initial injury severity often demonstrate markedly different functional outcomes over time [5]. Despite this variability, prognostic assessment in both clinical practice and research has traditionally relied on single time-point measures, most commonly the 90-day modified Rankin Scale (mRS). While widely accepted, such static assessments may not adequately capture the dynamic and evolving nature of neurological recovery [6], potentially limiting their ability to reflect long-term outcomes and patient-centered well-being.

Patients with moderate-to-severe ICH are frequently managed in the intensive care unit (ICU), where early neurological injury is compounded by critical illness–related physiological stress and exposure to invasive supportive interventions [7]. Previous studies have identified several early clinical factors—such as age, admission neurological status, hemorrhage volume, and intraventricular extension—as predictors of short-term outcomes or mortality after ICH [5,8]. However, the extent to which these early ICU-related factors shape long-term recovery trajectories, rather than single time-point outcomes, remains incompletely understood.

Recent methodological advances have enabled the identification of distinct functional recovery trajectories following acute neurological injury [9]. Trajectory-based approaches acknowledge that recovery is not uniform and allow patients to be classified into clinically meaningful recovery patterns over time. Emerging evidence in stroke populations suggests that such trajectories may better reflect long-term functional outcomes than single time-point assessments [10,11]. Nevertheless, whether trajectory-based classification provides incremental prognostic value beyond conventional single time-point measures has not been well established, particularly in ICU-treated ICH populations.

In addition, while functional status is commonly assessed using the mRS, functional outcomes alone may not fully capture patients’ long-term well-being [12]. Quality of life represents a critical patient-centered outcome after ICH, yet its relationship with longitudinal functional recovery patterns—and their relative prognostic performance—has not been well defined [13,14].

Therefore, we conducted a prospective cohort study of ICU-treated patients with intracerebral hemorrhage to (1) identify distinct functional recovery trajectories during the first year after hospital discharge, (2) examine the association between these trajectories and long-term quality of life, (3) determine early ICU clinical factors associated with trajectory membership, and (4) evaluate whether trajectory-based classification improves prediction of long-term QoL compared with conventional 90-day functional assessment. The present study aimed to characterize long-term functional recovery trajectories among ICU-treated patients with intracerebral hemorrhage, identify early clinical factors associated with trajectory membership, and evaluate the relationship between recovery trajectories and long-term quality of life. By focusing on longitudinal recovery patterns, this study seeks to improve early prognostic stratification following severe intracerebral hemorrhage.

## 2. Methods

### 2.1 Study Design and Setting

We conducted a prospective, longitudinal cohort study to characterize long-term functional recovery trajectories among patients with spontaneous ICH requiring ICU management and to examine early neurological and ICU-related clinical characteristics associated with trajectory membership. In addition, we evaluated the incremental prognostic value of trajectory-based classification compared with conventional single time-point functional assessment for predicting long-term quality of life.

Consecutive adult patients were enrolled between January 2024 and January 2025 in the neurological ICU of a tertiary academic hospital. Participants were followed prospectively for 12 months after hospital discharge, with predefined functional assessments at hospital discharge, 90 days, 6 months, and 12 months. The study was observational and designed to describe recovery patterns and associated factors without prespecifying causal relationships. Dichotomization was applied to facilitate clinically interpretable risk discrimination and receiver operating characteristic (ROC)-based comparison, while continuous analyses yielded consistent results. This study is reported in accordance with the Strengthening the Reporting of Observational Studies in Epidemiology (STROBE) Statement (**Supplementary Material**).

### 2.2 Participants and Enrollment

#### Data Collection and Outcome Assessment

Baseline demographic characteristics, vascular risk factors, comorbidities, and admission neurological status were collected prospectively using standardized case report forms. Neurological severity at ICU admission was assessed using the Glasgow Coma Scale (GCS). Hematoma volume was calculated from initial neuroimaging using the ABC/2 method, and the presence of intraventricular hemorrhage was recorded. The ICH score was calculated to provide a composite measure of initial disease severity.

ICU-related variables included requirement for mechanical ventilation, duration of continuous intravenous sedation (>48 hours vs ≤48 hours), vasopressor support, and ICU length of stay.

Functional outcomes were assessed using the modified Rankin Scale at hospital discharge and at 90 days, 6 months, and 12 months by trained assessors using standardized structured interviews.

Health-related quality of life at 12 months was assessed using the EuroQol 5-Dimension 5-Level questionnaire (EQ-5D-5L), a validated instrument widely used in stroke populations. The EQ-5D-5L index score ranges from values below 0 to 1, with higher scores indicating better perceived health status.

For prognostic analyses, 12-month QoL was dichotomized based on the cohort median EQ-5D-5L index score (0.72) to define favorable versus unfavorable outcomes.

Assessments were conducted by trained research personnel who were not involved in acute clinical management.

### 2.3 Statistical Analysis

Trajectory modeling was restricted to the 167 patients who survived to hospital discharge and completed all scheduled follow-up assessments. Longitudinal functional recovery trajectories were identified using latent class mixed modeling (LCMM) with an ordinal link function based on repeated mRS measurements at discharge, 90 days, 6 months, and 12 months. Trajectory analyses were restricted to patients who survived to hospital discharge and completed all scheduled follow-up assessments. Models with two to five latent classes were evaluated. Model selection was based on Bayesian Information Criterion (BIC), parsimony, minimum group size (>5%), mean posterior probability, and clinical interpretability. Model adequacy was evaluated using average posterior probabilities (≥0.70 considered acceptable), and patients were assigned to trajectory groups according to the maximum posterior probability rule. Models with two to five latent classes were estimated. The optimal model was selected according to BIC, parsimony, minimum group size (>5%), and clinical interpretability. Model adequacy was evaluated using average posterior probabilities (≥0.70 considered acceptable), and patients were assigned to trajectory groups according to the maximum posterior probability rule. To ensure a fair comparison, trajectory classification was treated as a summary representation of longitudinal functional evolution, while 90-day mRS represented the conventional single time-point assessment used in clinical practice.

After trajectory classification, baseline and ICU-related characteristics were compared across trajectory groups using appropriate parametric or nonparametric tests for continuous variables and chi-square or Fisher’s exact test for categorical variables.

Multicollinearity was assessed using variance inflation factors (VIFs), with values greater than 5 considered indicative of substantial multicollinearity. Covariates initially considered for multivariable modeling included age, sex, admission GCS score, hematoma volume, intraventricular hemorrhage, ICH score, mechanical ventilation, sedation duration (>48 hours), and ICU length of stay. To reduce model overfitting and avoid redundancy among highly correlated indicators of disease severity, the final model retained clinically essential variables selected a priori, including age, admission GCS score, hematoma volume, intraventricular hemorrhage, mechanical ventilation, and sedation duration. To reduce overfitting given the sample size, model complexity was restricted to clinically essential variables. Results are reported as odds ratios (ORs) with 95% confidence intervals (CIs). Given the available sample size and number of outcome groups, the final model was restricted to six clinically relevant predictors to reduce the risk of overfitting.

#### 2.3.1 Incremental Prognostic Analysis

To evaluate the prognostic performance of trajectory-based classification, ROC curve analyses were performed to assess discrimination for predicting 12-month QoL. The predictive ability of trajectory membership was compared with that of the conventional 90-day mRS.

Areas under the ROC curve (AUCs) were calculated and compared using the DeLong test. To further quantify incremental prognostic value, net reclassification improvement (NRI) and integrated discrimination improvement (IDI) were computed.

Trajectory membership was treated as an ordinal predictor (good, moderate, poor), while 90-day mRS was analyzed as a continuous variable. Higher predictive performance was defined by greater AUC values and statistically significant improvements in NRI and IDI.

#### 2.3.2 Additional Analyses

Sensitivity analyses were performed using alternative trajectory model specifications to assess robustness of the identified classes. Missing data were infrequent (<5%) and primarily involved baseline clinical variables. No variable had more than 3% missingness. Given the low proportion of missing data, complete-case analysis was used.

All analyses were conducted using R software (version 4.3, R Foundation for Statistical Computing, Vienna, Austria), and a two-sided *p* value < 0.05 was considered statistically significant.

## 3. Results

**Fig. 1.**
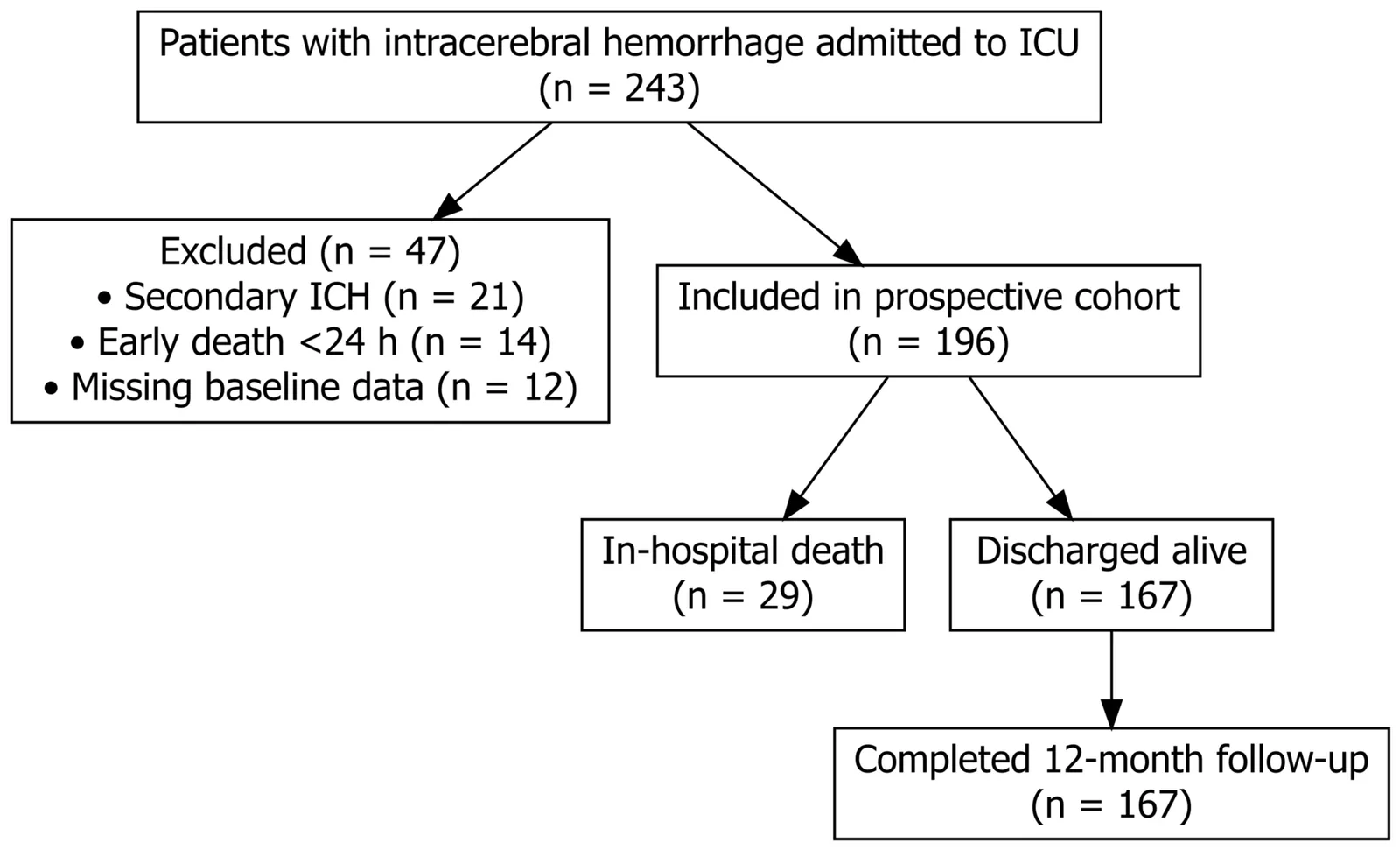
**Flow diagram of patient selection and follow-up in the ICU-based intracerebral hemorrhage cohort**. ICU, intensive care unit; ICH, intracerebral hemorrhage.

To describe the patient selection process and follow-up completion, all patients with intracerebral hemorrhage admitted to the intensive care unit during the study period were systematically screened (Fig. 1). Among 243 patients initially assessed for eligibility, 47 were excluded, including 21 with secondary intracerebral hemorrhage, 14 who died within 24 hours of admission, and 12 with missing baseline data. A total of 196 patients were therefore included in the prospective cohort. During the index hospitalization, 29 patients died, while 167 survived to hospital discharge; all surviving patients completed the scheduled 12-month follow-up. These results define a well-characterized ICU-based survivor cohort for the analysis of post-discharge functional recovery trajectories. Baseline comparisons between survivors and non-survivors are presented in **Supplementary Table 1**. Non-survivors exhibited substantially greater neurological severity, including lower admission GCS scores, larger hematoma volumes, and higher rates of intensive organ support.

Baseline demographic characteristics and ICU-related clinical features of the 196 included patients are summarized in Table 1. The mean age was 61.7 ± 12.4 years, and 60.2% of patients were male. Hypertension was the most common comorbidity (70.9%), followed by diabetes mellitus (23.5%) and prior stroke (15.8%). At ICU admission, the median Glasgow Coma Scale score was 11 (interquartile range [IQR], 8–14), and the median intracerebral hemorrhage volume was 27.6 mL (IQR, 14.3–42.8); 37.8% of patients had concomitant intraventricular hemorrhage. During the ICU stay, 52.0% of patients required mechanical ventilation, 44.4% received sedation for longer than 48 hours, and 32.1% required vasopressor support. The median ICU length of stay was 9.3 days (IQR, 5.8–14.6), and in-hospital mortality in the included cohort was 14.8% (29/196), reflecting substantial neurological severity and intensive supportive care requirements among enrolled patients.

**Table 1.** **Baseline and ICU characteristics of the study cohort (n = 196)**.

Characteristics	Overall cohort (n = 196)
Age, years, mean ± SD	61.7 ± 12.4
Male sex, n (%)	118 (60.2)
Hypertension, n (%)	139 (70.9)
Diabetes mellitus, n (%)	46 (23.5)
Prior stroke, n (%)	31 (15.8)
Admission GCS score, median (IQR)	11 (8–14)
ICH volume, mL, median (IQR)	27.6 (14.3–42.8)
Intraventricular hemorrhage, n (%)	74 (37.8)
Mechanical ventilation, n (%)	102 (52.0)
Sedation >48 h, n (%)	87 (44.4)
Vasopressor use, n (%)	63 (32.1)
ICU length of stay, days, median (IQR)	9.3 (5.8–14.6)
In-hospital mortality, n (%)	29 (14.8)
ICH score, median (IQR)	2 (1–3)
Follow-up completion at 12 months, n (%)	167 (85.2)

GCS, Glasgow Coma Scale; IQR, interquartile range.

Longitudinal mRS assessments conducted at discharge, 90 days, 6 months, and 12 months were used to characterize post-discharge functional recovery patterns. Trajectory analyses were restricted to the 167 patients who survived to hospital discharge and completed longitudinal follow-up assessments. Model comparison results are presented in **Supplementary Table 2**. Although the four-class model demonstrated a slightly lower BIC, the smallest class accounted for only a small proportion of the cohort. The three-class solution showed adequate class sizes and excellent classification accuracy and was therefore selected as the final model. The mean posterior probability for the selected three-class model was 0.91, indicating a high degree of classification certainty.

**Fig. 2.**
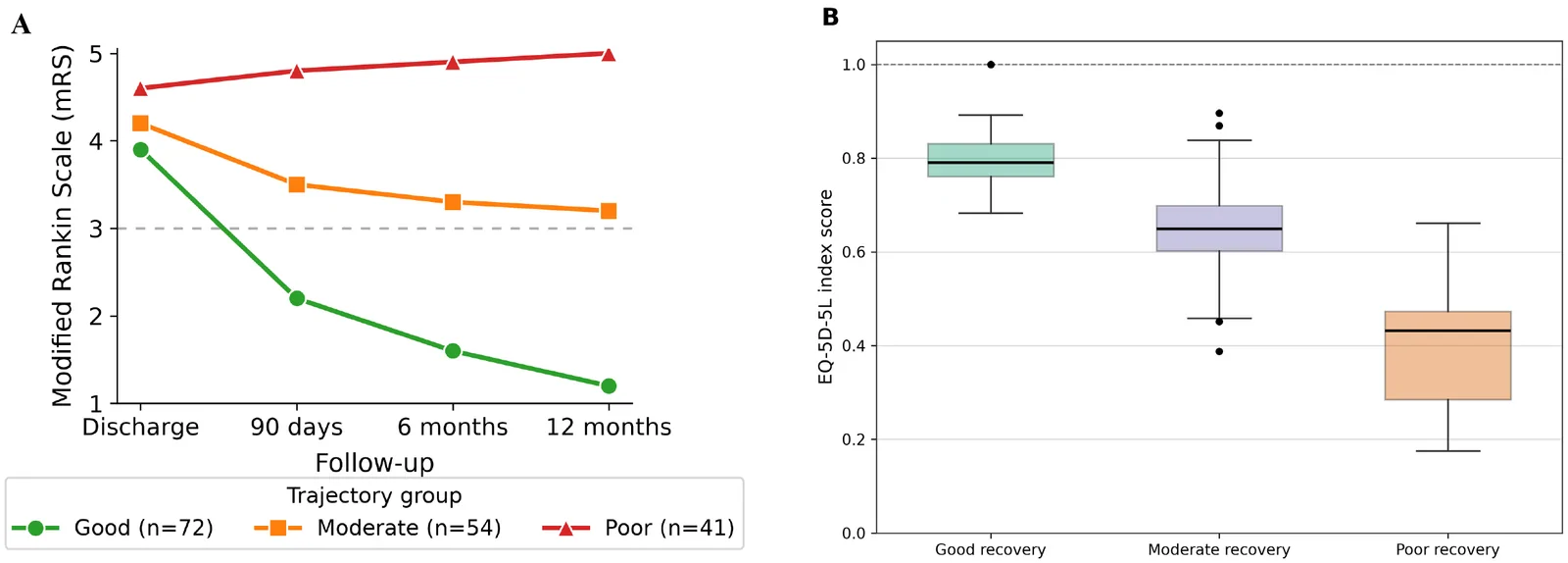
**Long-term functional recovery trajectories and associated quality of life after hospital discharge**. (A) Mean mRS trajectories over 12 months by recovery group. The y-axis is reversed so that upward movement indicates improvement. (B) Quality-of-life outcomes at 12 months according to functional recovery trajectories. The center line indicates the median, the box represents the IQR, and whiskers extend to 1.5 × IQR. Points beyond the whiskers are displayed as outliers. Sample sizes were: good recovery (n = 72), moderate recovery (n = 54), and poor recovery (n = 41). The horizontal dashed line at y = 1.0 indicates the upper bound of the EQ-5D utility scale (perfect health). mo, months.

LCMM identified three distinct functional recovery trajectories over time (Fig. 2A): a good recovery trajectory characterized by progressive improvement from moderate disability at discharge to persistently low mRS scores thereafter, a moderate recovery trajectory showing gradual but incomplete functional improvement, and a poor recovery trajectory marked by persistently high mRS scores with limited improvement. At 12 months, health-related quality-of-life scores differed across the three trajectories, demonstrating a graded pattern with the highest scores observed in the good recovery group and the lowest scores in the poor recovery group (Fig. 2B). The median EQ-5D-5L index score for the overall cohort was 0.72 (IQR, 0.51–0.86), which was used as the predefined cutoff for prognostic analyses. These findings indicate substantial heterogeneity in long-term functional recovery among ICU-treated ICH survivors.

Among the 167 hospital survivors included in trajectory modeling, 72 (43.1%) were classified into the good recovery trajectory, 54 (32.3%) into the moderate recovery trajectory, and 41 (24.6%) into the poor recovery trajectory. Baseline and ICU-related clinical characteristics were next compared across the three functional recovery trajectory groups (Table 2). Significant differences were observed among trajectory groups with respect to age, admission Glasgow Coma Scale score, intracerebral hemorrhage volume, prevalence of intraventricular hemorrhage, requirement for mechanical ventilation, duration of sedation exceeding 48 hours, and ICU length of stay (all *p* ≤ 0.01). Across worsening recovery trajectories, patients tended to be older, have lower admission GCS scores, larger hemorrhage volumes, a higher prevalence of intraventricular hemorrhage, and greater exposure to intensive ICU interventions. In contrast, sex distribution did not differ significantly among trajectory groups. These comparisons demonstrate systematic differences in baseline neurological severity and ICU clinical characteristics across long-term recovery trajectories.

**Table 2.** **Baseline and ICU characteristics according to functional recovery trajectories**.

Characteristics	Good recovery (n = 72)	Moderate recovery (n = 54)	Poor recovery (n = 41)	*p* value
Age, years	56.8 ± 11.2	62.9 ± 12.1	66.4 ± 12.7	<0.001
Male sex, n (%)	46 (63.9)	32 (59.3)	23 (56.1)	0.620
Admission GCS, median (IQR)	13 (11–15)	10 (8–13)	7 (5–10)	<0.001
ICH volume, mL	18.4 (10.6–28.9)	29.7 (18.2–44.6)	43.8 (29.5–62.4)	<0.001
Intraventricular hemorrhage, n (%)	16 (22.2)	21 (38.9)	23 (56.1)	<0.001
Mechanical ventilation, n (%)	27 (37.5)	30 (55.6)	27 (65.9)	0.010
Sedation >48 h, n (%)	21 (29.2)	26 (48.1)	24 (58.5)	0.006
ICU LOS, days	6.8 (4.6–10.2)	9.7 (6.3–14.8)	13.9 (9.2–18.6)	<0.001

LOS, length of stay.

Multivariable multinomial logistic regression analyses were performed using a prespecified parsimonious model including age, admission GCS score, hematoma volume, intraventricular hemorrhage, mechanical ventilation, and sedation duration. Variables such as sex, ICH score, and ICU length of stay were not retained in the final model because of limited incremental clinical information and potential overlap with other measures of disease severity (Table 3). Older age, lower admission Glasgow Coma Scale scores, and larger intracerebral hemorrhage volumes were associated with increased odds of membership in both the moderate and poor recovery trajectories, with consistently stronger associations observed for the poor recovery trajectory. Intraventricular hemorrhage, mechanical ventilation, and sedation exceeding 48 hours were significantly associated with membership in the poor recovery trajectory, whereas these variables were not significantly associated with the moderate recovery trajectory. Together, these results demonstrate that early neurological severity and selected ICU-related clinical features are differentially associated with long-term functional recovery trajectory membership among ICU-treated intracerebral hemorrhage survivors. Assessment of multicollinearity demonstrated no evidence of substantial collinearity among included variables, with all VIF values below 5.0.

**Table 3.** **Multinomial logistic regression analysis of early ICU factors associated with recovery trajectory membership**.

Predictor (early ICU)	Moderate vs good OR (95% CI)	*p* value	Poor vs good OR (95% CI)	*p* value
Age (per 10-year increase)	1.42 (1.11–1.83)	0.005	1.86 (1.38–2.51)	<0.001
Admission GCS (per point)	0.84 (0.77–0.92)	<0.001	0.71 (0.63–0.80)	<0.001
ICH volume (per 10 mL)	1.36 (1.12–1.65)	0.002	1.79 (1.41–2.26)	<0.001
Intraventricular hemorrhage	1.58 (0.82–3.04)	0.17	2.47 (1.26–4.86)	0.009
Mechanical ventilation	1.44 (0.78–2.64)	0.24	2.18 (1.13–4.21)	0.021
Sedation >48 h	1.62 (0.87–3.03)	0.13	2.31 (1.21–4.42)	0.012

OR, odds ratio; CI, confidence interval.

### Incremental Prognostic Value of Trajectory Classification

**Fig. 3.**
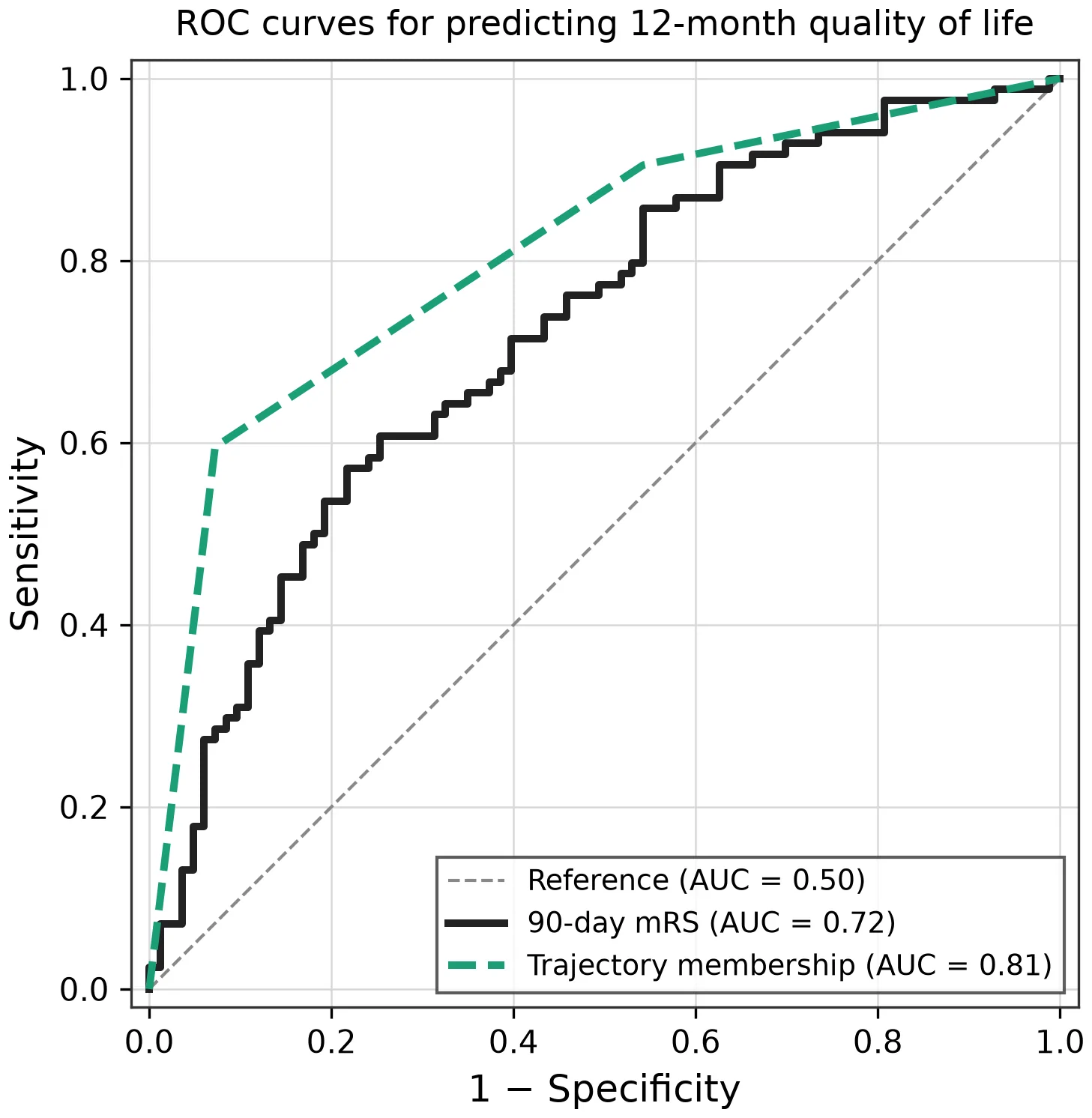
**ROC curve comparison for predicting 12-month quality of life**. ROC, receiver operating characteristic.

We next evaluated whether trajectory-based classification improved prediction of 12-month quality of life compared with conventional single time-point functional assessment using the 90-day mRS (Table 4, Fig. 3). The model incorporating 90-day mRS demonstrated moderate discrimination for predicting 12-month quality of life (AUC = 0.72; 95% CI, 0.64–0.79). In contrast, trajectory membership showed significantly improved discrimination (AUC = 0.81; 95% CI, 0.74–0.87), with a statistically significant difference between models (*p* = 0.003).

**Table 4.** **Incremental prognostic performance for predicting 12-month quality of life**.

Model	AUC (95% CI)	*p* value (vs 90-day mRS)	NRI	IDI
90-day mRS	0.72 (0.64–0.79)	—	—	—
Trajectory membership	0.81 (0.74–0.87)	0.003	0.27	0.09

mRS, modified Rankin Scale; AUC, area under the curve; NRI, net reclassification improvement; IDI, integrated discrimination improvement.

Trajectory-based classification further enhanced risk stratification, as reflected by a net reclassification improvement of 0.27 and an integrated discrimination improvement of 0.09. These findings indicate that longitudinal recovery trajectories provide incremental prognostic information beyond conventional single time-point outcome assessment.

## 4. Discussion

Beyond identifying the presence of heterogeneous recovery patterns, the present study demonstrated distinct differences in baseline neurological severity and long-term quality of life across the three trajectory groups.

### 4.1 Principal Findings

In this prospective ICU-based cohort of patients with spontaneous ICH, we identified three distinct and clinically meaningful long-term functional recovery trajectories over the first year following hospital discharge. These findings are consistent with prior trajectory-based analyses demonstrating heterogeneous recovery patterns after ICH rather than a uniform course of disability [10,15]. Our results extend this work by specifically focusing on ICU-treated patients, a subgroup underrepresented in previous trajectory studies. The clear graded association between trajectory membership and 12-month QoL further supports the clinical relevance of longitudinal recovery modeling. The three trajectory groups demonstrated a clear clinical gradient. Patients in the good recovery trajectory were generally younger, had higher admission GCS scores, smaller hematoma volumes, and fewer cases of intraventricular hemorrhage. In contrast, patients in the poor recovery trajectory exhibited greater neurological severity at presentation and experienced substantially worse long-term functional and quality-of-life outcomes. The moderate recovery trajectory represented an intermediate pattern between these two extremes. These findings support the clinical interpretability of the identified trajectory classes.

Importantly, beyond describing recovery heterogeneity, our study suggests that trajectory-based classification may provide additional prognostic information beyond conventional single time-point outcome assessment. Compared with the widely used 90-day mRS, trajectory membership showed improved discrimination for predicting long-term QoL, suggesting that longitudinal recovery patterns capture clinically meaningful information not reflected in isolated measurements. These findings support a shift from static outcome assessment toward dynamic recovery modeling in ICH.

Beyond confirming heterogeneity, our findings suggest that divergence in recovery trajectories may emerge early during the acute critical care phase, when secondary injury mechanisms and systemic stress responses are most active. This supports a dynamic view of recovery in which long-term functional phenotypes reflect not only initial hemorrhagic injury but also the biological and clinical processes occurring during ICU management.

### 4.2 Heterogeneity of Recovery in ICU-Treated ICH

Recovery after ICH is increasingly recognized as a dynamic process extending well beyond the traditional 90-day endpoint. Previous longitudinal studies have shown that meaningful functional improvement may continue up to 12 months post-injury [11,16,17]. Moreover, trajectory-based approaches have demonstrated that patients cluster into distinct recovery patterns, including sustained improvement, delayed recovery, and persistent severe disability [10,15,18].

However, most prior cohorts included mixed-severity or population-based samples. ICU-treated patients represent a higher-risk subgroup characterized by more severe neurological injury and greater exposure to critical care interventions. Long-term mortality and disability remain substantial among ICH survivors [18,19,20,21], and outcomes following mechanical ventilation for ICH are particularly poor [20,22].

From a mechanistic perspective, this subgroup may be uniquely susceptible to amplified secondary injury cascades. Primary hematoma-induced damage initiates neuroinflammation, perihematomal edema formation, oxidative stress, and blood–brain barrier disruption. In critically ill patients, these processes may be compounded by systemic inflammation, hypoxia, metabolic instability, and hemodynamic fluctuations inherent to severe illness. Such systemic stressors may interfere with neuronal survival, synaptic reorganization, and adaptive neuroplasticity, thereby contributing to early divergence into distinct recovery trajectories.

Collectively, these findings support a conceptual framework in which recovery trajectories represent emergent phenotypes arising from the interaction between primary brain injury and critical illness–related systemic stress, rather than fixed consequences of initial hemorrhage severity alone.

### 4.3 Trajectory-Based Prognostication Beyond Single Time-Point Outcomes

Traditional prognostic approaches in intracerebral hemorrhage have largely relied on single time-point outcome measures, most commonly the 90-day mRS. While clinically practical, such static assessments may fail to capture the dynamic and evolving nature of neurological recovery.

In the present study, trajectory-based classification improved prediction of long-term QoL compared with 90-day mRS, indicating that longitudinal modeling provides additional prognostic information beyond conventional approaches. By integrating repeated functional assessments over time, trajectory modeling captures both the direction and tempo of recovery, which are not reflected in isolated outcome measurements.

These findings have important implications for prognostic modeling in neurocritical care. Rather than conceptualizing outcome as a fixed state at a predefined time point, recovery after ICH should be understood as a dynamic process characterized by evolving functional trajectories. Incorporating trajectory-based approaches into prognostic frameworks may therefore enhance risk stratification and better align predictions with patient-centered outcomes. Because trajectory classification incorporates repeated functional assessments over time, it inherently contains more longitudinal information than a single 90-day mRS measurement. Therefore, the observed improvement in predictive performance should be interpreted within this context and does not necessarily imply superiority in prospective clinical prediction. However, trajectory membership was derived from multiple longitudinal mRS assessments, whereas the comparator model relied solely on a single 90-day mRS measurement. Therefore, the superior predictive performance of trajectory classification may partly reflect the incorporation of additional longitudinal information rather than the trajectory framework itself.

### 4.4 Relationship Between Recovery Trajectories and Quality of Life

A central observation of this study is the strong graded association between functional recovery trajectories and 12-month QoL. Prior research has demonstrated that long-term QoL after ICH is closely related to functional status [3,23] and may follow its own trajectory over time [13]. Our results align with these findings and further indicate that longitudinal functional patterns are meaningfully linked to patient-reported well-being. A stepwise decline in EQ-5D-5L scores was observed across the good, moderate, and poor recovery trajectories, indicating that trajectory membership reflects meaningful differences in long-term patient-centered outcomes rather than statistical classifications alone.

Importantly, psychological and behavioral sequelae after hemorrhagic stroke, including emotional dysregulation and apathy, may independently influence QoL beyond motor disability [12,14]. In ICU-treated patients, prolonged sedation, mechanical ventilation, and critical illness–related encephalopathy may further increase vulnerability to cognitive dysfunction and delirium, which have been associated with persistent neurocognitive impairment. These factors may contribute to poorer long-term outcomes; however, the underlying mechanisms cannot be determined from the present study.

Thus, patients with persistent severe disability trajectories may experience compounded impairments affecting motor, cognitive, and psychosocial domains. Trajectory-based classification may therefore better capture the multidimensional lived experience of survivors compared with conventional single time-point assessments. The median EQ-5D-5L value used for dichotomization was broadly consistent with thresholds previously associated with substantial post-stroke disability and reduced health-related quality of life, supporting the clinical relevance of the prognostic classification used in this study.

### 4.5 Early Neurological Severity and ICU-Related Factors

Consistent with established prognostic literature, older age, lower admission Glasgow Coma Scale scores, larger hematoma volumes, and intraventricular hemorrhage were associated with poorer recovery trajectories. These factors are well-recognized predictors of adverse outcome following ICH [24,25], and hematoma characteristics remain central components of validated prognostic models such as the max-ICH score [8,26,27].

Beyond baseline neurological injury, ICU-related variables were associated with trajectory membership. Mechanical ventilation and prolonged sedation were significantly associated with membership in the poor recovery trajectory. However, these ICU-related variables should be interpreted cautiously because they may primarily reflect greater neurological injury, overall disease severity, and clinical complexity rather than exerting independent effects on recovery. Furthermore, ICU management decisions are influenced by multiple factors beyond hemorrhage volume and admission neurological status, including anatomical hemorrhage location, neurosurgical interventions, comorbidity burden, and other clinical considerations that were not comprehensively captured in the present study.

The observed associations may indicate that patients requiring more intensive ICU support represent a subgroup with more severe neurological and systemic illness. However, the present observational design does not allow determination of whether ICU-related exposures directly influence recovery trajectories.

Although causality cannot be inferred from observational data, these findings suggest that ICU-related variables may serve as markers of clinical severity and identify patients at increased risk for unfavorable recovery trajectories. Further studies incorporating more comprehensive clinical data are needed to clarify these relationships.

### 4.6 Clinical Implications

The identification of distinct recovery trajectories has several practical implications. First, trajectory-based classification may improve prognostic accuracy beyond conventional 90-day outcome assessment, supporting more individualized patient counseling. Second, early identification of patients at risk for unfavorable recovery trajectories may inform targeted follow-up and rehabilitation strategies, particularly for ICU survivors at high risk of long-term disability. Third, incorporating trajectory-based modeling into clinical research may provide a more nuanced framework for evaluating treatment effects compared with traditional dichotomized endpoints.

### 4.7 Strengths and Limitations

This study has several strengths, including prospective design, standardized longitudinal functional assessment, and application of trajectory modeling in an ICU-specific cohort. Importantly, we extended prior work by demonstrating the incremental prognostic value of trajectory-based classification beyond conventional outcome measures.

Nonetheless, several limitations should be acknowledged. First, analyses were restricted to hospital survivors, introducing potential survivorship bias. Second, this was a single-center study conducted in a highly selected cohort of ICU-treated intracerebral hemorrhage survivors. Therefore, the identified recovery trajectories and associated prognostic relationships may not be directly generalizable to broader intracerebral hemorrhage populations, including patients with milder disease, non-ICU management, or different healthcare systems. Third, although key markers of initial severity were adjusted for, residual confounding cannot be excluded. Fourth, while the modified Rankin Scale is widely validated, it may not fully capture cognitive or psychosocial impairments that contribute to long-term recovery heterogeneity. In addition, outcome assessors were not formally blinded to baseline clinical characteristics and ICU exposures, which may have introduced a degree of measurement bias.

An important limitation is survivorship bias, as trajectory analyses were restricted to patients who survived to hospital discharge. As shown in **Supplementary Table 1**, non-survivors had substantially greater baseline severity. Therefore, the identified trajectories primarily reflect recovery patterns among hospital survivors and may not be generalizable to all patients with severe intracerebral hemorrhage. Although the proportion of missing data was low, complete-case analysis may have introduced a small degree of bias if data were not missing completely at random.

In addition, several potentially important determinants of ICU management and long-term recovery, including acute kidney injury, hemorrhage location, neurosurgical intervention, comorbidity burden, rehabilitation exposure, cognitive impairment, depression, and post-discharge care, were unavailable and therefore could not be incorporated into the analyses [28]. Consequently, residual confounding may have influenced the observed associations between ICU-related variables and recovery trajectory membership. From a clinical perspective, the primary value of trajectory classification may lie in improving early prognostic stratification rather than guiding specific ICU interventions. Identification of patients at risk for poor recovery trajectories may facilitate individualized follow-up planning, communication with families, and allocation of post-acute care resources. In addition, the EQ-5D-5L index was dichotomized using the cohort median for prognostic analyses. Although this approach facilitated ROC-based comparisons, it may have resulted in some loss of information and should not be interpreted as representing a clinically established quality-of-life threshold. Trajectory classification should be interpreted primarily as a framework for characterizing recovery heterogeneity and long-term prognostic patterns rather than as a real-time clinical prediction tool. Future studies are needed to determine whether trajectory membership can be predicted earlier using acute clinical characteristics and early follow-up data. Although the selected model demonstrated high average posterior probabilities, some degree of classification uncertainty is inherent to latent class modeling and may have resulted in limited misclassification of trajectory membership.

## 5. Conclusions

In this prospective ICU-based cohort of patients with spontaneous intracerebral hemorrhage, we identified three distinct and clinically meaningful long-term functional recovery trajectories over the first year following hospital discharge. These trajectories were closely aligned with quality of life and were systematically associated with early neurological severity and ICU-related clinical characteristics. Importantly, trajectory-based classification provided incremental prognostic value beyond conventional single time-point outcome assessment. These findings support the integration of longitudinal trajectory-based approaches into prognostic modeling and long-term care planning for critically ill patients with ICH.

## Data Availability

The data underlying this study are quantitative clinical and follow-up outcome data collected prospectively from patients with intracerebral hemorrhage treated in the intensive care unit. De-identified data are not publicly available because the hospital rules limited it, but may be made available from the corresponding author upon reasonable request and with appropriate institutional approvals.
