# Supplementary material for: Trajectory-Based Modeling Improves Long-Term Outcome Prediction Beyond 90-Day Assessment in ICU-Treated Intracerebral Hemorrhage
Source: Rev Neurol. 2026 Aug 25;81(8):52441. doi: 10.31083/RN52441 (PMC13541425; doi:10.31083/RN52441)
Supplement: Supplementary file 1 [file 1576-6578-81-8-52441-s1.zip › Supplementary Tables.docx]

### Supplementary Table S1. **Baseline Characteristics of Survivors and Non-Survivors During Index Hospitalization**

| **Variable** | **Survivors (n=167)** | **Non-survivors (n=29)** | **P value** |
| --- | --- | --- | --- |
| Age, years | 60.3 ± 11.8 | 69.8 ± 13.1 | <0.001 |
| Male sex, n (%) | 102 (61.1) | 16 (55.2) | 0.55 |
| Admission GCS score, median (IQR) | 12 (9–14) | 5 (3–8) | <0.001 |
| ICH score, median (IQR) | 2 (1–3) | 4 (3–5) | <0.001 |
| ICH volume, mL, median (IQR) | 24.8 (13.6–39.2) | 58.4 (41.7–78.5) | <0.001 |
| Intraventricular hemorrhage, n (%) | 56 (33.5) | 18 (62.1) | 0.004 |
| Mechanical ventilation, n (%) | 77 (46.1) | 19 (65.5) | <0.001 |
| Sedation >48 h, n (%) | 70 (41.9) | 23 (79.3) | <0.001 |

### Supplementary Table S2. Model Fit Statistics for Latent Class Mixed Models With Two to Five Trajectory Classes

| **Classes** | **BIC** | **Smallest Class (%)** | **Mean Posterior Probability** |
| --- | --- | --- | --- |
| 2 | 3928.4 | 33.2 | 0.88 |
| 3 | 3875.2 | 24.6 | 0.91 |
| 4 | 3872.9 | 7.4 | 0.84 |
| 5 | 3886.7 | 3.1 | 0.77 |
